# The adenosine analog prodrug ATV006 is orally bioavailable and has preclinical efficacy against parental SARS-CoV-2 and variants

**DOI:** 10.1126/scitranslmed.abm7621

**Published:** 2022-05-17

**Authors:** Liu Cao, Yingjun Li, Sidi Yang, Guanguan Li, Qifan Zhou, Jing Sun, Tiefeng Xu, Yang Yang, Ruyan Liao, Yongxia Shi, Yujian Yang, Tiaozhen Zhu, Siyao Huang, Yanxi Ji, Feng Cong, Yinzhu Luo, Yujun Zhu, Hemi Luan, Huan Zhang, Jingdiao Chen, Xue Liu, Renru Luo, Lihong Liu, Ping Wang, Yang Yu, Fan Xing, Bixia Ke, Huanying Zheng, Xiaoling Deng, Wenyong Zhang, Chuwen Lin, Mang Shi, Chun-Mei Li, Yu Zhang, Lu Zhang, Jun Dai, Hongzhou Lu, Jincun Zhao, Xumu Zhang, Deyin Guo

**Affiliations:** ^1^ Centre for Infection and Immunity Studies (CIIS), School of Medicine, Shenzhen Campus of Sun Yat-sen University, Guangdong 518107, China.; ^2^ Shenzhen Key Laboratory of Small Molecule Drug Discovery and Synthesis, Department of Chemistry, College of Science, Academy for Advanced Interdisciplinary Studies, Southern University of Science and Technology, Shenzhen, Guangdong 518055, China; ^3^ Medi-X Pingshan, Southern University of Science and Technology, Shenzhen, Guangdong 518118, China; ^4^ State Key Laboratory of Respiratory Disease, National Clinical Research Center for Respiratory Disease, Guangzhou Institute of Respiratory Health, the First Affiliated Hospital of Guangzhou Medical University, Guangzhou, Guangdong 510182, China; ^5^ Guangzhou Laboratory, Bio-island, Guangzhou, Guangdong 510320, People's Republic of China.; ^6^ Shenzhen Key Laboratory of Pathogen and Immunity, National Clinical Research Center for infectious disease, State Key Discipline of Infectious Disease, Shenzhen Third People's Hospital, Second Hospital Affiliated to Southern University of Science and Technology, Shenzhen, Guangdong 518112, China; ^7^ Guangzhou Customs District Technology Center, Guangzhou, Guangdong 510623, China.; ^8^ Guangdong Province Key Laboratory of Laboratory Animals, Guangdong Laboratory Animals Monitoring Institute, Guangzhou, Guangdong 510663, China.; ^9^ School of Medicine, Southern University of Science and Technology, Shenzhen, Guangdong 518055, China; ^10^ Center for Disease Control and Prevention of Guangdong Province, Guangzhou, Guangdong 511430, China

## Abstract

Severe acute respiratory syndrome coronavirus 2 (SARS-CoV-2), the virus driving the ongoing coronavirus disease 2019 (COVID-19) pandemic, continues to rapidly evolve. Due to the limited efficacy of vaccination in prevention of SARS-CoV-2 transmission and continuous emergence of variants of concern (VOC), orally bioavailable and broadly efficacious antiviral drugs are urgently needed. Previously we showed that the parent nucleoside of remdesivir, GS-441524, possesses potent anti-SARS-CoV-2 activity. Herein, we report that esterification of the 5′-hydroxyl moieties of GS-441524 markedly improved antiviral potency. This 5′-hydroxyl-isobutyryl prodrug, ATV006, demonstrated excellent oral bioavailability in rats and cynomolgus monkeys and exhibited potent antiviral efficacy against different SARS-CoV-2 VOCs in vitro and in three mouse models. Oral administration of ATV006 reduced viral loads and alleviated lung damage when administered prophylactically and therapeutically to K18-hACE2 mice challenged with the Delta variant of SARS-CoV-2. These data indicate that ATV006 represents a promising oral antiviral drug candidate for SARS-CoV-2.

## Teaser

ATV006 is an orally bioavailable antiviral with efficacy against SARS-CoV-2 infection in preclinical models.

## INTRODUCTION

The outbreak of the coronavirus disease 2019 (COVID-19) pandemic, caused by severe acute respiratory syndrome coronavirus 2 (SARS-CoV-2), has been ongoing for over two years and has resulted in over 440 million confirmed infections and over 6 million reported deaths worldwide as of 4 March 2022 (*1*). SARS-CoV-2 is a positive-sense, single-stranded RNA virus belonging to the genus *Betacoronavirus* of the family *Coronaviridae* (*2*). Two other members of the same genus, namely severe acute respiratory syndrome coronavirus (SARS-CoV) and Middle East respiratory syndrome coronavirus (MERS-CoV), have caused outbreaks with substantial fatality rates in 2003 and 2012, respectively (*3*, *4*). Given the repeated and accelerating emergence of highly pathogenic coronaviruses, it is increasingly important to develop broadly effective anti-coronaviral agents to combat the pandemics of COVID-19 and the future emerging coronaviruses.

Although the coronavirus has a certain proofreading ability (*5*), it still has a high mutation rate. Progressive mutational change in the virus is therefore inevitable, which leads to the emergence of new variants. At present, the World Health Organization (WHO) has classified many variants of concern (VOC) of SARS-CoV-2 (*6*), such as the Alpha (B.1.1.7), Beta (B.1.351), Delta (B.1.671.2), and Omicron (B.1.1.529) variants. The Omicron and Delta variants are highly contagious and have rapidly become dominant, accounting for more than 99% of recent SARS-CoV-2 infections. The Delta variant may lead to vaccine breakthrough infections associated with higher viral load and longer duration of shedding (*7*–*9*). The Omicron variant has higher transmission capacity and exhibits stronger ability to evade protection conferred by prior infection and currently available vaccines (*10*–*13*); however, the symptoms caused by the Omicron variant are thought to be reduced compared to the Delta variant (*14*, *15*). Therefore, with the current limited protection of vaccines against these SARS-CoV-2 variants, there is an urgent need to develop orally available and broadly efficacious anti-coronaviral agents.

The RNA-dependent RNA polymerase (RdRp), a key component of the viral replication and transcription machinery, is considered a primary target for development of anti-SARS-CoV-2 drugs (*16*–*19*). Several nucleoside or nucleotide analogs, including remdesivir, molnupiravir (EIDD-2801), favipiravir and AT-527, originally developed by targeting the RdRp of other RNA viruses, have been repurposed for SARS-CoV-2 (*20*–*24*). Among them, remdesivir is the first approved drug by the US Food and Drug Administration (FDA) for the treatment of patients with COVID-19 (*25*, *26*), and it has been shown to reduce the risk of hospitalization or death by 87% when administered early to non-hospitalized, high-risk patients (*27*). Molnupiravir, as well as a main protease inhibitor, Paxlovid, were granted emergency use authorization (EUA) by the FDA for the treatment of non-hospitalized COVID-19 patients (*28*, *29*). However, AT-527 and favipiravir did not meet primary endpoint in clinical trials for patients with mild to moderate disease (*30*, *31*).

Although remdesivir is efficacious in the treatment of those with mild disease at high risk for COVID-19 progression (*27*), its administration by intravenous (IV) injection limits its use in clinical settings (*25*, *32*, *33*). We and others reported that the parent nucleoside of remdesivir, GS-441524 (with CAS registry number 1191237-69-0, also known as 69-0), potently inhibited SARS-CoV-2 infection in cell culture and mouse models (*19*, *34*, *35*). GS-441524 is a 1’-cyano-substituted adenosine analog with broad-spectrum antiviral activities across multiple virus families (*36*–*39*). Thus, GS-441524 may serve as a lead compound to develop drug candidates for oral delivery. Recently, two derivatives of GS-441524 (GS-621763 and VV116) with improved oral bioavailability and anti-SARS-CoV-2 activity have been reported (*40*–*42*).

In this study, we reported a series of ester prodrugs of GS-441524 with improved antiviral potency. The 5′-isobutyryl derivative ATV006, demonstrated improved oral pharmacokinetic (PK) profiles and potently inhibited the replication of SARS-CoV-2 and its variants. In three different animal models, ATV006 could suppress infection and pathogenesis of SARS-CoV-2. These results indicate that ATV006 is a promising drug candidate for further clinical development for COVID-19.

## RESULTS

### Design and synthesis of prodrugs of adenosine analogs that target SARS-CoV-2 RNA polymerase.

**Fig. 1.**
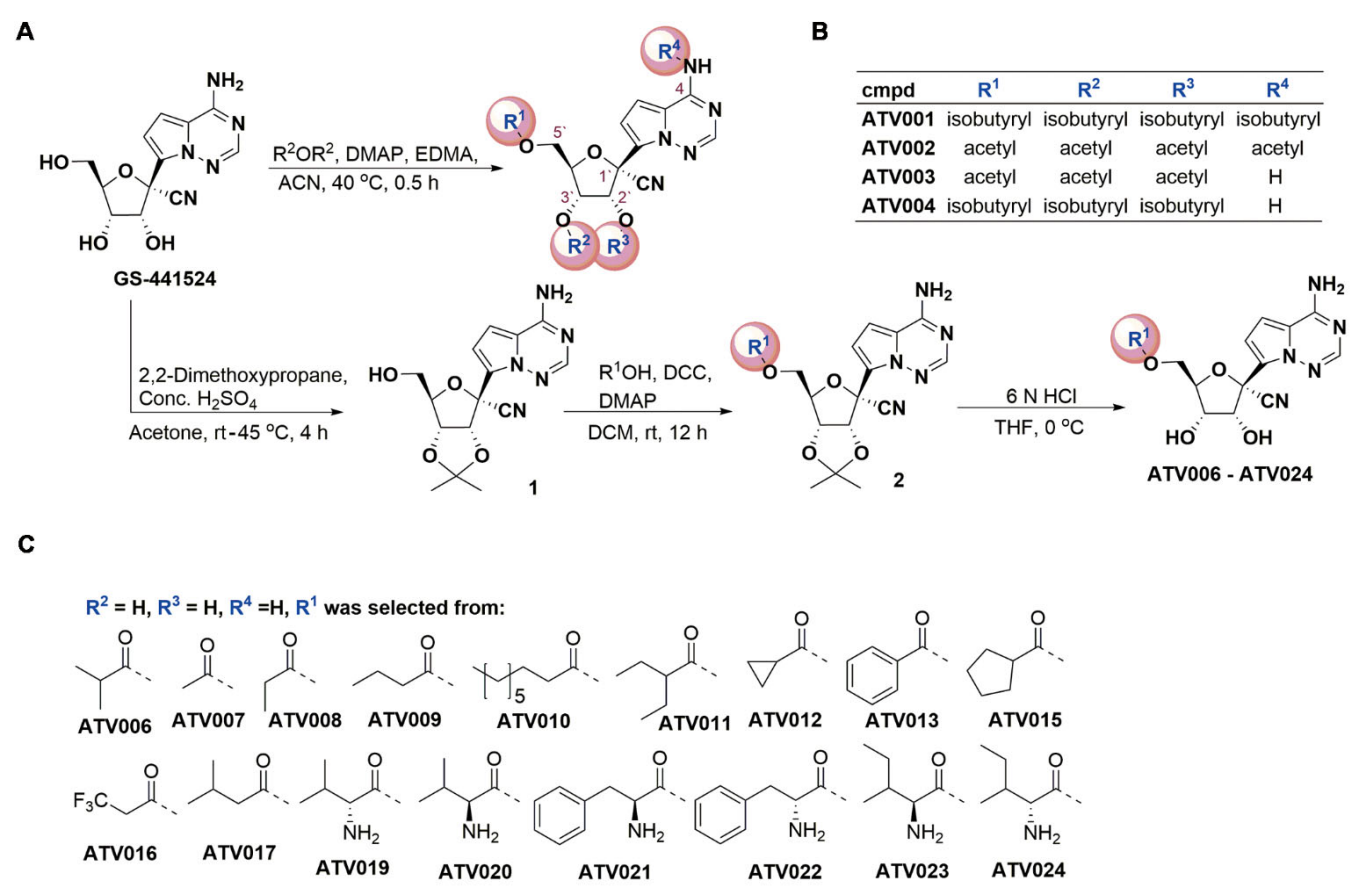
The chemical structure and synthesis of GS-441524-derived prodrugs. (**A**) The synthetic process of GS-441524 derivatives is shown. R^2^OR^2^, Acid anhydrides with carbonyl of R^2^; rt, room temperature; DCC, Dicyclohexylcarbodiimide; THF, Tetrahydrofuran. (**B**) Compounds (cmpd) ATV001 to ATV004 were synthesized from GS-441524 by one-step acylation reactions with related acid anhydride in the presence of 4-dimethylaminopyridine (DMAP) and ethylene dimethacrylate (EDMA). (**C**) For the synthesis of ATV006-024, 2′,3′-hydroxyl moieties of GS-441524 were protected with acetonide by using 2,2-dimethoxypropane in the presence of sulfuric acid (H_2_SO_4_). Different aliphatic acids, aromatic acids, or amino acids were reacted with intermediate 1 to product the corresponding esters by DCC/DMAP-mediated condensation, respectively. The subsequent hydrolysis reaction of compound 2 with 6N hydrochloric acid (HCl) yielded desired compounds ATV006 to ATV024.

Although the adenosine analog GS-441524 was effective against SARS-CoV-2 in vitro and in mouse models, as shown in our previous study, it suffers from poor oral bioavailability, which hampers its further development as an oral drug (*34*). To overcome this limitation, we devoted our efforts to synthesizing GS-441524 prodrugs by employing short-chain fatty acids (SCFAs) or amino acids to mask the polar hydroxyl- or amino-groups. For this, 21 compounds with different substitutions at the positions of R^1^, R^2^, R^3^ or R^4^ of GS-441524 were designed and chemically synthesized (Fig. 1). The antiviral effect of the compounds was initially evaluated using a SARS-CoV-2 replicon system (pBAC-SARS-CoV-2-Replicon-Luc), established in our previous work (*43*), which carries all the genes essential for SARS-CoV-2 replication, including RdRp, and the luciferase reporter gene, but does not produce infectious virus particles. Promising candidates with improved potency and bioavailability were empirically selected for further testing with live SARS-CoV-2 in cell culture and mouse models after considering their antiviral activity, cytotoxicity, pharmacokinetic (PK) properties as well as synthetic cost (fig. S1).

We first tested the percentage inhibition in the replicon system (fig. S2A) and then selected 17 compounds with inhibition rate higher than 90% at 10 μM to measure their concentration for 50% of maximal effect (EC_50_), which ranged from 0.217 to 2.351 μM (fig. S2B and table S1). The compounds ATV001 and ATV002 with isobutyryl amide or acetyl amide at the base moiety showed decreased antiviral activities (fig. S2A and B), probably due to the biostable amide group that obstructed hydrogen bond formation between the inhibitor and the RNA template (*44*, *45*). Tri-acetyl esterification of the hydroxyl groups on C5′ (R^1^), C2’ (R^2^) and C3′ (R^3^) positions (ATV003) did not substantially change the activity, whereas the tri-isobutyryl on C5′ (R^1^), C2’ (R^2^) and C3′ (R^3^) (ATV004), which has the same structure to GS-621763 (*41*, *42*), and mono-isobutyryl-modification of 5′-hydroxyl group (ATV006) improved the inhibitory activities in the replicon system in comparison with its parent GS-441524 (fig. S2B and table S1). The compounds ATV007-024 contained a R^1^ group modified with straight, cyclic, or branched SCFAs, benzyl acyl-groups, or amino acid-groups. Some of these compounds displayed an improvement in potency relative to GS-441524. Six compounds (ATV019-024) bearing L- or D- amino acid ester were designed to improve drug absorption by targeting the peptide transporter family 1 (PepT1) (*46*). However, these compounds did not show improved activity against the replicon or improved permeability in Caco-2 cells (table S2). Together, SCFA esterification at the C5′ position could improve the potency relative to GS-441524. Therefore, we selected the top six compounds ranked by low to high EC_50_ in replicon system for further analysis of anti-SARS-CoV-2 activity with live virus, including different variants, in cell culture models.

### The adenosine analog prodrug ATV006 potently inhibits the replication of authentic SARS-CoV-2 and its variants of concern.

**Fig. 2.**
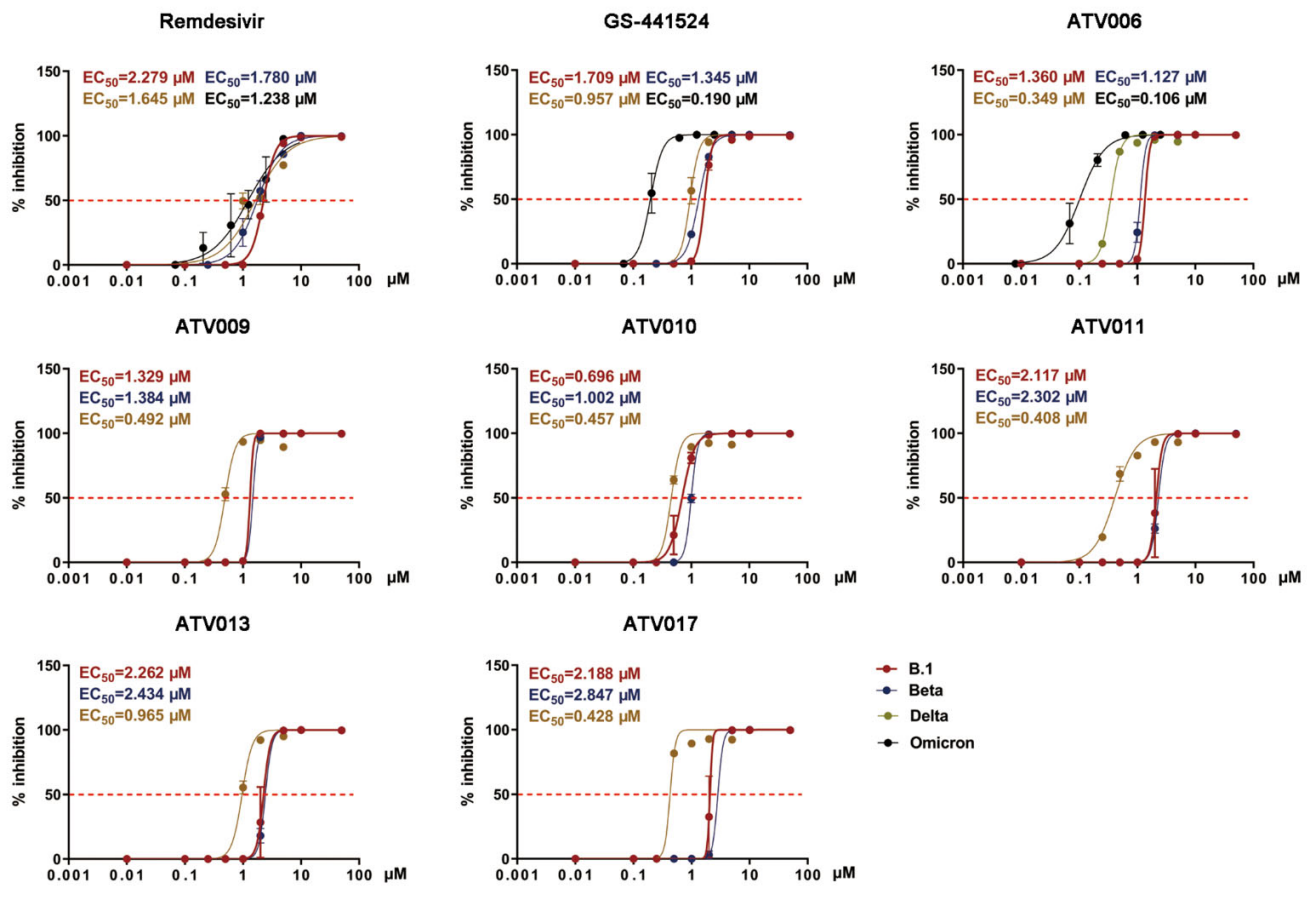
GS-441524 derivatives exhibit antiviral activity against SARS-CoV-2 variants in vitro. Vero E6 cells were infected with different strains of SARS-CoV-2(B.1, Beta, Delta and Omicron) at a multiplicity of infection (MOI) of 0.05 and treated with dilutions of the indicated compounds for 48 hours. Viral yield in the cell supernatant was then quantified by qRT-PCR [% inhibition= (viral RNA copies of treatment group / viral RNA copies of placebo control group) × 100]. The values of the concentration for 50% of maximal effect (EC_50_) for each compound are shown above each plot and indicated with the red dashed line.

The antiviral efficacy of the six SCFA prodrugs (ATV006, ATV009-011, ATV013 and ATV017) was evaluated in Vero E6 cells infected with different strains of SARS-CoV-2, including the early strain, B.1, and three SARS-CoV-2 VOC variants, Beta, Delta, and Omicron (Fig. 2, Table 1, and table S3). The compounds showed improved potency against SARS-CoV-2 as compared to remdesivir and GS-441524, which was consistent with the results of the SARS-CoV-2 replicon system (Fig. 2 and Table 1). Among them, ATV010 exhibited low micromolar EC_50_ value with early strain B.1 and Beta variant. In contrast, ATV006 had an overall >4-fold and >12-fold potency improvement in inhibiting the replication of Delta and Omicron variants, with EC_50_ values reaching 0.349 and 0.106 μM, respectively. The compounds exhibited similar antiviral activity in Huh7 cells to that in Vero E6 cells (fig. S3 and table S4). Together, these results indicate that 5`OH esterified compounds could potently inhibit the replication of SARS-CoV-2 and its variants.

**Table 1.** Anti-SARS-CoV-2 activity and cytotoxicity of adenosine analog prodrugs in comparison with remdesivir and GS-441524.

	EC_50_ (μM) in Vero E6 cells					CC_50_ (μM)
Compound	SARS-CoV-2 (B.1)	SARS-CoV-2 (Beta, B.1.351)	SARS-CoV-2 (Delta, B.1.617.2)		SARS-CoV-2 (Omicron, B.1.1.529)	
Remdesivir	2.279	1.780	1.645		1.238	>50
GS-441524	1.709	1.354	0.957		0.190	>50
ATV006	1.360	1.127	0.349		0.106	128.00
ATV009	1.329	1.484	0.492			>50
ATV010	0.696	1.002	0.457			44.62
ATV011	2.117	2.302	0.408			>50
ATV013	2.262	2.434	0.965			>50
ATV017	2.188	2.847	0.428			>50

The cytotoxicity of the compounds was evaluated on Vero E6 cells with the CCK8 assays (fig. S4). The results showed that most of the compounds had low toxicity with the concentration of 50% cytotoxicity (CC_50_) values greater than 50 μM, except for ATV010, which had a CC_50_ value of 44.62 μM, indicating an overall low toxicity. The therapeutic index (CC_50_/EC_50_) of ATV006 tested with Omicron variant was as high as 1207.55 in Vero E6 cells. Considering the potent inhibition against Delta and Omicron variants, high selectivity against cell proliferation, moderate LogP value (0.86) and better water solubility (686.02 μg/mL) (table S5), ATV006 was selected for further studies.

### ATV006 has favorable pharmacokinetic profiles in rats and cynomolgus monkeys.

**Fig. 3.**
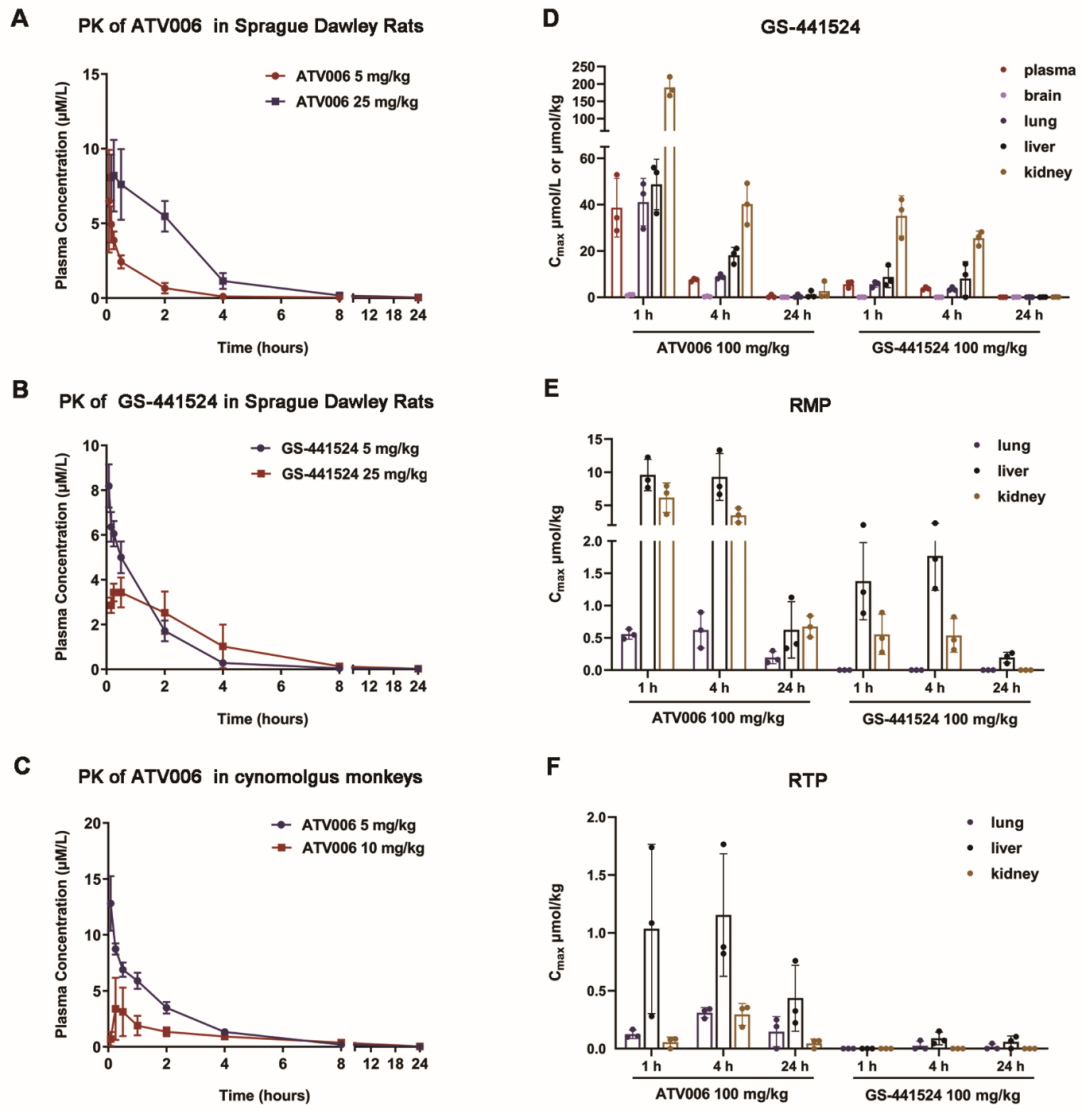
Pharmacokinetic profile of ATV006 and GS-441524 in Sprague Dawley rats and cynomolgus monkeys. (**A**) A time-plasma concentration curve is shown for the nucleoside GS-441524 following a single IV administration of ATV006 (5 mg/kg) or oral administration (25 mg/kg) to Sprague Dawley rats (n = 3, mean ± SD). (**B**) A time-plasma concentration curve is shown for the nucleoside GS-441524 following a single IV administration of GS-441524 (5 mg/kg) or oral administration (25 mg/kg) to Sprague Dawley rats (n = 3, mean ± SD). (**C**) A time-plasma concentration curve is shown for the nucleoside GS-441524 following a single IV administration of ATV006 (5 mg/kg) or oral administration (10 mg/kg) to cynomolgus monkeys (n = 3, mean ± SD). (**D to F**) The tissue distribution of GS-441524 (D), remdesivir nucleotide monophosphate (RMP) (E), and remdesivir nucleotide triphosphate (RTP) (F) is shown for plasma, brain, lung, liver, and kidney after oral administration of 100 mg/kg ATV006 or GS-441524 to Sprague Dawley rats (n = 3, mean ± SD).

To assess the oral absorption of ATV006, PK studies were conducted in Sprague Dawley rats, using GS-441524 as a control. Following oral dosing of 25 mg/kg in rats, ATV006 displayed high oral bioavailability (F %) of 81.5%, whereas GS-441524 had poor oral bioavailability with a F% value of 21.7% (Table 2 and 3), the latter being similar to the data from the National Center for Advancing Translational Sciences (NCATS) of National Institutes of Health (NIH) (*47*). The maximum blood concentration (C_max_) of 8.2 μM was achieved 0.5 hours after the oral administration of ATV006, indicating its effective blood exposure to be 3.8 to 30-fold higher than the concentration for 90% of maximal effect (EC_90_) of ATV006 (Fig. 3A and Table 2). The C_max_ of 3.4 μM was achieved 1 hour after oral administration of GS-441524, indicating that the absorption time of GS-441524 is longer and the final concentration is lower when compared with ATV006 (Fig. 3B and Table 3). In cynomolgus monkeys, the average C_max_ of 3.71 μM was reached at 1.5 hours after the oral administration of 10 mg/kg ATV006 and the plasma concentration decayed with a half-life (T_1/2_) of 4 hours (Fig. 3C and Table 2). The oral bioavailability of ATV006 was about 30% in macaques (Table 2), which had an apparent increase when compared with the 8.3% of GS-441524 previously shown by NCATS of NIH (*47*). As a compound with an oral bioavailability of greater than 10% has the potential for development as an oral drug (*48*), ATV006 well met such standard as an oral drug candidate for further testing in animal models.

**Table 2.** Pharmacokinetic profile of ATV006 in Sprague-Dawley rats and cynomolgus monkeys. ATV006 was administered by the indicated route, and the parameters were calculated based on the LC-MS/MS analysis of the parent nucleoside, GS-441524 (n=3 per group).

	rat		monkey	
parameters	IV (5 mg/kg)	Oral (25 mg/kg)	IV (5 mg/kg)	Oral (10 mg/kg)
AUC_last_ (μM*h)	5.6 ± 1.6	22.8 ± 4.6	20.46 ± 1.68	12.22 ± 0.84
T_1/2_ (h)	1.5 ± 0.2	1.2 ± 0.1	1.78 ± 0.6	4.08 ± 0.94
T_max_ (h)		0.5 ± 0.25	0.083	1.50 ± 2.20
C_max_ (μM)	8.7 ± 7.8	8.2 ± 0.8	12.82 ± 2.43	3.71 ± 2.24
F (%)		81.5 ± 15.6%		30.08%

**Table 3.** Pharmacokinetic profile of GS-441524 in Sprague-Dawley rats. GS-441524 was administered by the indicated route, and the parameters were calculated based on the LC-MS/MS analysis of GS-441524 (n=3 per group).

	rat	
parameters	IV (5 mg/kg)	Oral (25 mg/kg)
AUC_last_ (μM*h)	11.0 ± 1.7	11.7 ± 5.1
T_1/2_ (h)	1.2 ± 0.1	1.4 ± 0.2
T_max_ (h)		1.00 ± 0.3
C_max_ (μM)	10.7 ± 1.5	3.4 ± 0.5
F (%)		21.7 ± 9.7%

Next, we explored the tissue distribution of a single oral dose of ATV006 and GS-441524 at 100 mg/kg in rats by measuring its parent nucleoside GS-441524 and its monophosphate and triphosphate forms (Fig. 3, D to F and Table 4). The results revealed that oral administration of ATV006 achieved broad distribution in the plasma, liver, and kidneys as well as in the lung, the major target organ for SARS-CoV-2 infection. In contrast, after oral administration of GS-441524, the concentrations of GS-441524, remdesivir nucleotide monophosphate (RMP), and remdesivir nucleotide triphosphate (RTP) in various tissues were lower than that of ATV006 (Fig. 3, D to F). Likewise, oral administration of ATV006 showed extensive distribution in the plasma, liver, kidney and lung of C57BL/6 mice (fig. S5). Together, the PK results showed that ATV006 had improved oral bioavailability and tissue distribution relative to GS-441524.

**Table 4.** PK parameters of GS-441524, RMP, and RTP in rat plasma and tissues after oral administration of ATV006 or GS-441524 100 mg/kg.

Administration	Compound	Source	T_max_	C_max_	AUC_last_
			(h)	(μmol/L or μmol/kg)	(h*μmol/L or h*μmol/kg)
ATV006	GS-441524	plasma	1.00	38.687	126.172
		brain	1.00	1.019	4.335
		lung	1.00	41.071	142.339
		liver	1.00	48.716	239.494
		kidney	1.00	190.013	661.392
	RMP	lung	4.00	0.620	9.376
		liver	1.00	9.579	97.243
		kidney	1.00	6.147	51.416
	RTP	lung	4.00	0.310	5.966
		liver	4.00	1.154	18.553
		kidney	4.00	0.295	3.205
GS-441524	GS-441524	plasma	1.00	5.632	31.294
		brain	24.0	0.073	0.822
		lung	1.00	5.601	34.327
		liver	1.00	8.776	124.688
		kidney	1.00	35.165	199.702
	RMP	lung		0	0
		liver	4.00	1.767	19.680
		kidney	1.00	0.554	1.913
	RTP	lung	4.00	0.024	0.418
		liver	4.00	0.088	1.562
		kidney		0	0

### Orally administered ATV006 could effectively suppress SARS-CoV-2 replication in mouse models.

**Fig. 4.**
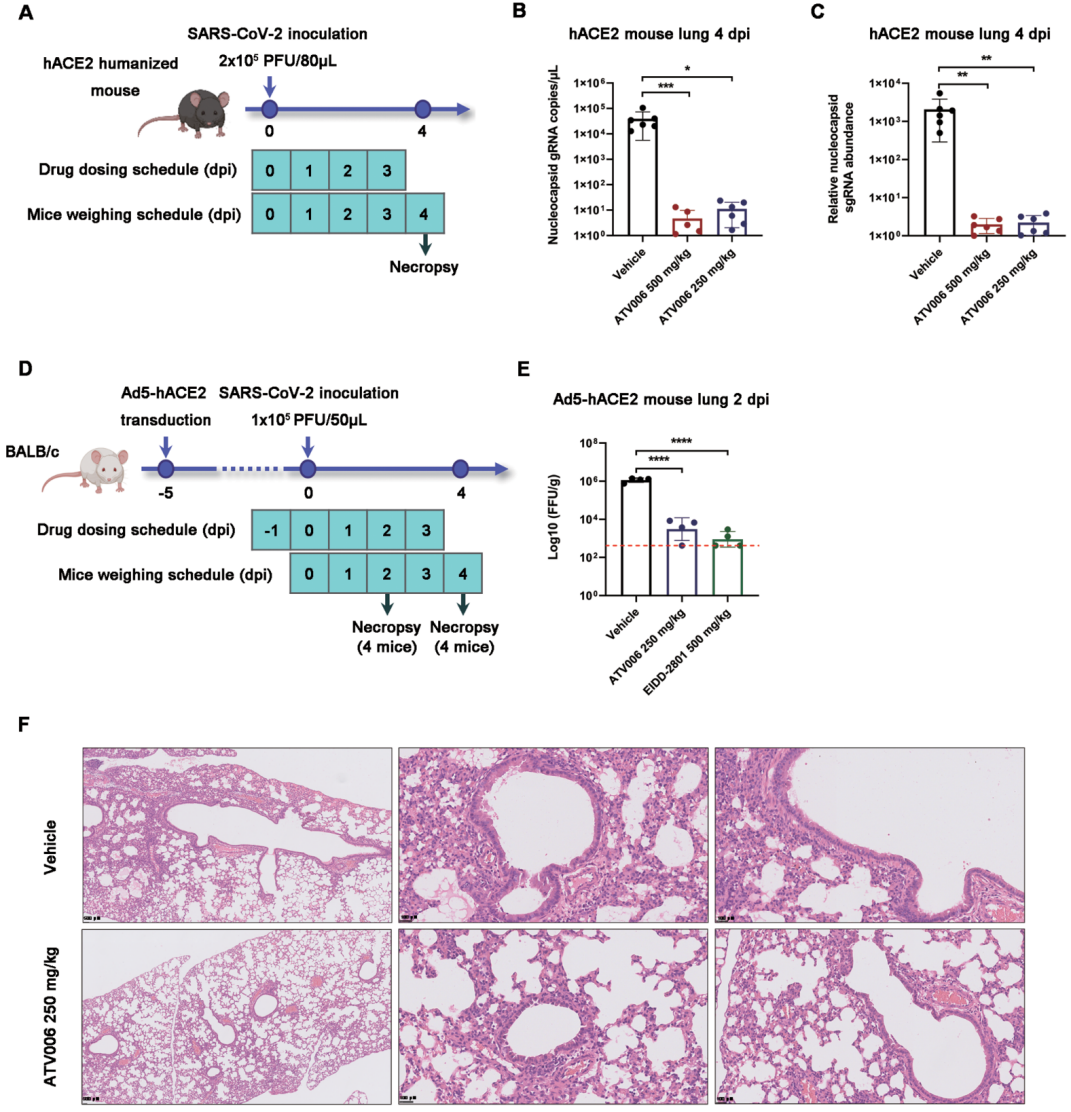
**ATV006 treatment reduces viral load and prevents lung pathology in hACE2 knock-in and Ad5-hACE2 mouse models. (A)** Experimental timeline for SARS-CoV-2 infection in hACE2 humanized mice. Mice were intranasally inoculated with the B.1 strain of SARS-CoV-2 (2 × 10^5^ PFU per mouse) and were treated with vehicle (control), ATV006 (250 mg/kg, orally, once daily), or ATV006 (500 mg/kg, orally, once daily) starting at the time of infection (n=6 mice per group). (**B and C**) Viral load was measured in the lungs at 4 dpi by qRT-PCR analysis of nucleocapsid (N) gRNA (B) and sgRNA (C). The limit of detection of qRT-PCR was 0.5 copies/μL. (**D**) Experimental timeline for SARS-CoV-2 infection in Ad5-hACE2 mice. Ad5-hACE2-transduced mice infected with B.1 strain of SARS-CoV-2 (1 × 10^5^ PFU per mouse) were treated with vehicle (control), ATV006 (250 mg/kg, orally, once daily), or EIDD-2801 (500 mg/kg, orally, once daily) (n=8 mice per group). (**E**) Viral titers were measured in the lungs of Ad5-hACE2 mice at 2 dpi by focus forming assay (FFA) and reported as focus-forming units (FFU) per gram of lung tissue. The red dashed line in (E) indicates the limit of detection. (**F**) Histopathology analysis is shown for lungs isolated at 4 dpi from SARS-CoV-2 infected Ad5-hACE2 mice treated with vehicle or ATV006 (250 mg/kg). Data are presented as mean ± SD. Statistical analysis was conducted using one-way ANOVA with Dunnett’s correction for multiple comparisons (lung viral sgRNA and lung virus titer) or Kruskal-Wallis test with Dunn's correction for multiple comparisons (lung viral gRNA). ∗p ≤ 0.05; ∗∗p ≤ 0.005; ∗∗∗p ≤ 0.0005; ∗∗∗∗p ≤ 0.0001.

We next investigated the antiviral activity of orally administered ATV006 in two different mouse models of SARS-CoV-2 (strain B.1), one with knock-in of humanized angiotensin-converting enzyme 2 (KI-hACE2) at the *mAce2* gene locus (*49*) and the other with adenovirus-delivered human ACE2 (Ad5-hACE2) (*50*). The KI-hACE2 mice were intranasally inoculated with SARS-CoV-2 and treated with ATV006 or vehicle control (Fig. 4A). To better determine the degree of SARS-CoV-2 replication, we detected both the genomic RNA (gRNA) and subgenomic RNA (sgRNA), the latter being produced by discontinuous synthesis and representing a biomarker of coronavirus replication (*51*, *52*) (fig. S6). In the control group, both SARS-CoV-2 gRNA and sgRNA reached high concentrations in the lung, indicating that the mouse infection model was well-established. In contrast, SARS-CoV-2 gRNA and sgRNA were hardly detectable at 4 days post infection (dpi) in the ATV006 treatment groups (Fig. 4, B and C), demonstrating robust inhibition of SARS-CoV-2 replication by ATV006.

We further tested the antiviral potency of ATV006 in the Ad5-hACE2 mouse model, which supports SARS-CoV-2 infection and pathogenesis in the mouse lung (*50*). The mice were treated vehicle, ATV006, or EIDD-2801 as a positive control (Fig. 4D). The results showed that ATV006 (250 mg/kg) and EIDD-2801 (500 mg/kg) could reduce the viral load and pathological damage of the lung (Fig. 4, E and F). Together, ATV006 showed potent anti-SARS-CoV-2 efficacy in different mouse models.

### Prophylactic ATV006 treatment reduces lung damage and protects K18-hACE2 mice.

**Fig. 5.**
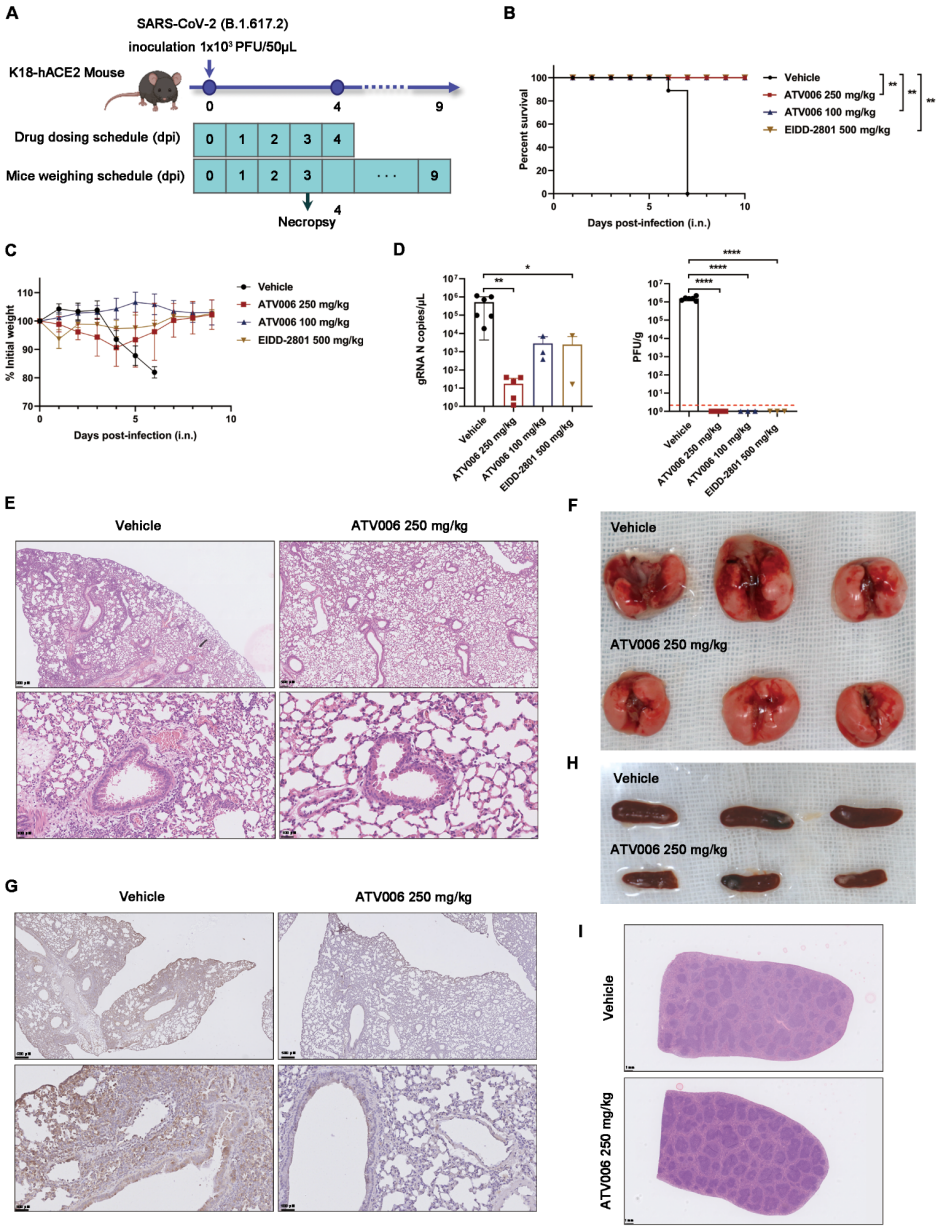
Prophylactic ATV006 treatment is efficacious against SARS-CoV-2 in K18-hACE2 mice. (**A**) The experimental timeline is shown. K18-hACE2 mice were intranasally inoculated with the SARS-CoV-2 Delta variant (1 × 10^3^ PFU per mouse) and treated with vehicle (control, n=11), ATV006 (250 mg/kg, orally, once daily, n=11), ATV006 (100 mg/kg, orally, once daily, n=8) or EIDD-2801 (500 mg/kg, orally, once daily, n=8). (**B**) The survival curve is shown. (**C**) Change in body weight was measured over time post infection. (**D**) Viral load from lung tissue at 3 dpi was analyzed by qRT-PCR and plaque assay. The detection limit of qRT-PCR was 0.5 copies/μL. The red dashed line in (D, right panel) indicates the limit of detection for the plaque assay. (**E to G**) Histopathology (E), gross pathology (F), and immunohistochemistry detection of SARS-CoV-2 S protein (G) are shown for lungs isolated from the indicated treatment groups. S protein is denoted by brown staining in (G). (**H and I**) Gross pathology (H) and histopathology (I) are shown for spleens isolated from the indicated treatment groups. Data in (C and D) are presented as mean ± SD. Statistical analysis was conducted using a Log-Rank test (survival), a one-way ANOVA with Dunnett’s correction for multiple comparisons (lung titer), or a Kruskal-Wallis test with Dunn's correction for multiple comparisons (lung viral RNA). ∗p ≤ 0.05; ∗∗p ≤ 0.005; ∗∗∗∗p ≤ 0.0001.

We next tested the antiviral potency of ATV006 in the widely used K18-hACE2 transgenic mouse model, which is highly susceptible to SARS-CoV-2 infection and can lead to death of infected mice (*53*). In a prophylactic model, the mice were challenged with the Delta variant (Fig. 5A). In this model, the control group gradually lost weight beginning on 4 dpi and died starting on the 6 dpi; all control mice died by 7dpi, but all mice in the treatment groups survived (Fig. 5, B and C). At 3 dpi, we evaluated the abundance of SARS-CoV-2 virus in mouse lung through quantitative real-time polymerase chain reaction (qRT-PCR) and plaque assay. The amount of viral RNA of the treatment groups was lower than that of the control group, with ATV006 (250 mg/kg) group significantly reducing viral RNA compared to the control (p=0.002, Fig. 5D). For the ATV006 (250 or 100 mg/kg) and EIDD-2801 (500 mg/kg) group, virus titers were reduced below the limit of detection in plaque assays at 3 dpi (Fig. 5D). Histopathological analyses were performed with the lungs of the mice infected with SARS-CoV-2 at 3 dpi. The vehicle-treated mice showed evidence of pathology, including inflammatory cell infiltration ranging from the trachea and peri-alveolar space to the interstitium; in contrast, ATV006-treated animals had reduced evidence of lung pathology (Fig. 5, E and F). Immunohistochemistry staining showed that the spike (S) protein of SARS-CoV-2 was hardly detectable at 3 dpi in the lungs of mice treated with ATV006 (250 mg/kg) (Fig. 5G). Compared with the ATV006 treatment group, the spleen of the mice in the control group was enlarged, and the white pulp was atrophied to varying degrees (Fig. 5, H and I). Furthermore, ATV006 reduced the production of inflammatory cytokines and chemokines in the lung tissues (table S6). Together, these results demonstrated that ATV006 possesses robust anti-SARS-CoV-2 efficacy in a mouse model of prophylactic treatment.

### Therapeutic administration of ATV006 reduces virus titers and lung damage caused by Delta variant infection in K18-hACE2 mice.

**Fig. 6.**
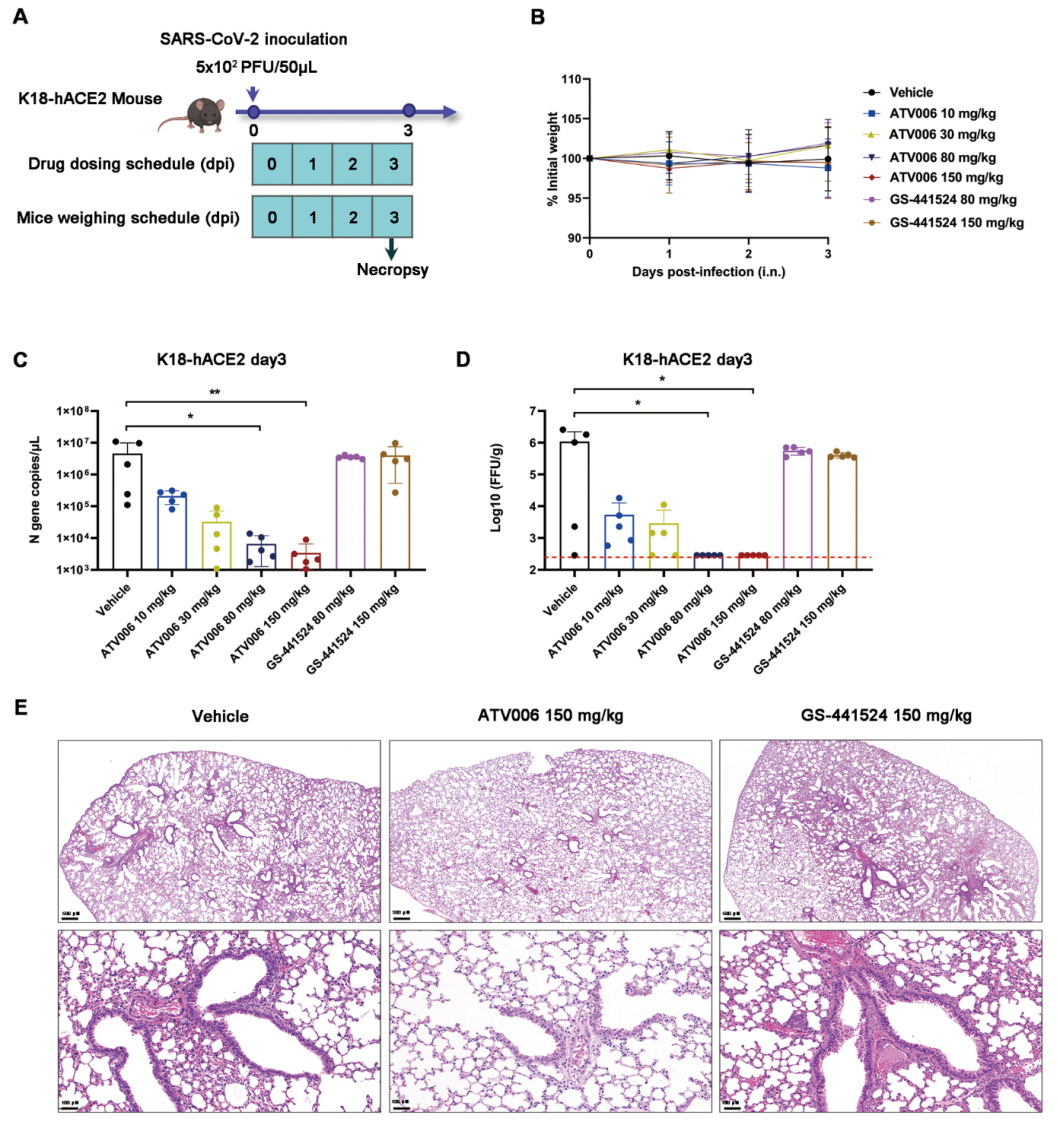
Therapeutic ATV006 treatment is efficacious against SARS-CoV-2 in K18-hACE2 mice. (**A**) The experimental timeline is shown. K18-hACE2 mice were intranasally inoculated with the SARS-CoV-2 Delta variant (5 × 10^2^ PFU per mouse) and were treated with vehicle (control, n=5), ATV006 (at 10, 30, 80, or 150 mg/kg orally, BID, n=5), or GS-441524 (at 80 or 150 mg/kg orally, BID, n=5). (**B**) Change in body weight was measured over time post infection. (**C and D**) Viral titers from lungs tissue at 3 dpi were analyzed by qRT-PCR (C) and focus-forming assay (FFA) (D). The detection limit of qRT-PCR was 0.5 copies/μL. The red dashed line in (D, right panel) indicates the limit of detection for the FFA. (**E**) Histopathology is shown for lungs isolated from mice in the indicated treatment groups. Data in (B to D) are presented as mean ± SD. Statistical analysis was conducted using Kruskal-Wallis tests with Dunn's correction for multiple comparisons (lung viral RNA, lung titer). ∗p ≤ 0.05; ∗∗p ≤ 0.005.

Next, we tested the therapeutic antiviral efficacy of ATV006 in K18-hACE2 mice (Fig. 6A). The body weight of the mice in each group did not change over the first three days post inoculation of the virus (Fig. 6B), which resembled the previous results (Fig. 5B). At 3 dpi, we evaluated the abundance of SARS-CoV-2 virus in mouse lung through qRT-PCR and plaque assay (Fig. 6, C and D). The mice treated with ATV006 at different dosages had reduced viral load in the lungs and the effect was in a dose-dependent manner. Viral titer in the high ATV006 dosage groups (80 and 150 mg/kg) was reduced below the detection limit at 3 dpi (Fig. 6D). In contrast, treatment with either 80 mg/kg or 150 mg/kg of GS-441524 did not inhibit virus replication in this model. Histopathological analyses were performed with the lung tissue isolated from infected mice at 3 dpi. ATV006 (150 mg/kg)-treated mice alleviated the symptoms in the lungs, except for evidence of a small amount of hemorrhage, whereas GS-441524 (150 mg/kg) and vehicle-treated mice showed multiple sites of injury and inflammatory cell infiltration (Fig. 6E).

Together, our results showed that oral administration of ATV006 could efficiently inhibit SARS-CoV-2 replication and ameliorate SARS-CoV-2-induced lung disease in mouse models of both prophylactic and therapeutic treatment. Prophylactic dosing of ATV006 could prevent the death of the K18-hACE2 mice challenged with the Delta variant of SARS-CoV-2. These results demonstrated the potential of ATV006 as an orally bioavailable anti-SARS-CoV-2 drug.

## DISCUSSION

COVID-19, caused by SARS-CoV-2, is still spreading globally, threatening human health and economic development. Vaccine-induced or naturally-acquired protective immunity necessary to disrupt transmission chains had been hampered by the rapid evolution and emergence of VOCs (*8*–*13*, *54*). Therefore, effective and broad-spectrum anti-SARS-CoV-2 drugs are desperately needed to treat patients with COVID-19 with breakthrough infections. These infections are increasingly common in vaccinated populations and are driven by VOCs which can evade pre-existing immunity. As COVID-19 is an acute infectious disease, antiviral treatment can exert its best effect at the early stage of the infection. In contrast, at the late stage of COVID-19 in patients who are hospitalized, anti-inflammatory therapy may be more beneficial in lessening the symptoms. Therefore, orally bioavailable anti-SARS-CoV-2 drugs suitable for outpatients are superior to injectable drugs applied to hospitalized patients.

Among the three drugs currently approved by FDA for the treatment of COVID-19, remdesivir and molnupiravir target the viral RdRp for their antiviral activity. However, the obligatory IV administration of remdesivir limits its clinical application (*25*). Previously, we reported GS-441524, the major metabolite and the parent nucleotide of remdesivir, has a better inhibitory activity on SARS-CoV-2 and mouse hepatitis virus (MHV)-A59 (*34*). However, the unfavorable oral PK prevented its further development into an oral drug (*34*, *47*). To address this issue, we synthesized a series of SCFA and amino acid prodrugs of GS-441524 aiming at overcoming these limitations. Among the compounds synthesized, the isobutyryl adenosine analog ATV006 improved oral absorption and potently inhibited the replication of SARS-CoV-2, including the Delta and Omicron variants. Furthermore, compared to remdesivir, ATV006 is structurally simpler and easier to synthesize by a three-step transformation with GS-441524 as starting material, which may reduce the cost and accelerate mass production.

After administration of ATV006, it is rapidly hydrolyzed by cellular esterases to produce the parent nucleoside GS-441524 (*55*, *56*), which then undergoes three steps of phosphorylation and is transformed to the active triphosphate form, the same active component as that of remdesivir and GS-441524. Therefore, ATV006 and remdesivir have the same mechanism of stalling SARS-CoV-2 polymerase by targeting viral RdRp (*45*, *57*–*59*). It is expected that ATV006 could be active against different variants of SARS-CoV-2, as the RdRp is the highly conserved amongst coronaviruses. Indeed, our current study demonstrated that ATV006 has a broad antiviral activity against different variants of SARS-CoV-2. The parent nucleoside GS-441524 was previously reported to be broadly active against viruses that belong to the families *Paramyxoviridae, Coronaviridae,* and *Filoviridae* (*36*–*39*, *60*), indicating the potential for more broad antiviral application of ATV006.

We tested the anti-SARS-CoV-2 efficacy of ATV006 with cell culture infection and three different mouse models by four independent research groups at three different BSL-3 facilities, and it was noted that the anti-SARS-CoV-2 activity of ATV006 was robust and consistent. Among the three mouse models (knock-in hACE2, Ad5-hACE2 and K18-hACE2) (*49*, *50*, *53*), the highly sensitive K18-hACE2 mice were used both in prophylactic and therapeutic models. Although K18-hACE2 mice can suffer fatal viral encephalitis from the neuroinvasion of SARS-CoV-2 (*61*) and the metabolites of ATV006 were not detectable in the mouse brain, ATV006 could effectively prevent the death of K18-hACE2 mice infected by SARS-CoV-2 Delta variant in the prophylactic model. One possible explanation for such potent efficacy is that prophylactic administration of ATV006 may effectively block SARS-CoV-2 replication early after infection and consequently avoid the neuroinvasion of SARS-CoV-2.

However, our study has several limitations. First, SARS-CoV-2 mouse models cannot fully recapitulate human infection and COVID-19 pathogenesis. For example, K18-hACE2 mice can suffer fatal viral encephalitis from neuroinvasion of SARS-CoV-2 in addition to pneumonia (*61*). Therefore, the antiviral effect of ATV006 shown in mouse models needs to be tested in non-rodent preclinical models and ultimately in human clinical trials. Second, the oral bioavailability of ATV006 was tested in both rat and macaque models, but due to limitations of non-human primate experiments, the oral availability of ATV006 was not compared head-to-head with its parent nucleoside, GS-441524, in macaques. Third, in the therapeutic model of ATV006, we only tested the dosing scheme with first oral administration at 6 hours post infection and only with treatment twice a day for three days. Future studies are needed to explore the latest dosing time and lowest dosages of ATV006 that are efficacious. Lastly, although we have evaluated the antiviral activity of ATV006 against different SARS-CoV-2 variants (including an early strain, B.1, as well as Beta, Delta, and Omicron), the broad-spectrum antiviral effect of ATV006 on other coronaviruses (including other human and animal coronaviruses) was not tested.

Collectively, our results demonstrated that ATV006 is orally bioavailable and has potent efficacy against SARS-CoV-2. This efficacy extended to variants of SARS-CoV-2. These data suggest that ATV006 represents a promising drug candidate for the treatment for COVID-19 and potentially for emerging coronavirus diseases in the future.

## MATERIALS AND METHODS

### Study Design

The aim of this study was to discover new antiviral compounds that possess potent anti-SARS-CoV-2 activity and can be administered orally. For this purpose, we designed and synthesized a series of derivatives of GS-441524, the parent nucleoside analog of remdesivir, by employing short-chain fatty acid (SCFAs) or amino acid modifications to mask the polar hydroxyl- or amino-groups. We first measured the antiviral activity of these compounds using a SARS-CoV-2 replicon system and further evaluated their anti-SARS-CoV-2 activity with live virus, including the early strain, B.1, and the Beta, Delta and Omicron variants in Vero-E6 and Huh7 cell cultures. Cytotoxicity was assessed in the Vero-E6 cell lines. In vitro experiments were repeated at least twice. By considering the anti-SARS-CoV-2 activity, selectivity index, LogP value and water solubility of the compounds, ATV006 was selected for further studies. Pharmacokinetic (PK), tissue distribution, and efficacy studies in mice, rats, and macaques were intended to gain the data for the oral bioavailability, druggability and in vivo anti-SARS-CoV-2 activity. Three SARS-CoV-2 mouse models were used in this study, including the mice with adenovirus-delivered hACE2 (Ad5-hACE2), knock-in hACE2 at the *mAce2* gene locus (KI-hACE2) and transgenic K18-hACE2. Mice were age-, sex-, and genetic background-matched and randomized to different groups of at least three animals per group before the start of each experiment. The preventive and therapeutic effects of ATV006 were studied by weight monitoring, virus detection (qRT-PCR, focus forming assay, plaque assay or immunostaining analysis), histopathology and survival analysis. Sample size calculation and blinding were not performed.

### Compounds, cells, and viruses

The compounds presented here (ATV001-024) were synthesized at our in-house facility, analyzed by ^1^H nuclear magnetic resonance spectroscopy (NMR), ^13^C NMR and high-resolution mass spectrometer (HRMS) and purified by high performance liquid chromatography (HPLC) with a purity of >95%. HEK 293T, Caco-2 and Huh7 cells were obtained from American Tissue Culture Collection (ATCC). The African green monkey kidney Vero E6 cell line (Vero E6) was kindly provided by Hui Zhang (Sun Yat-sen University). HEK 293T, Vero E6, Caco-2 and Huh7 cells were cultured in Dulbecco’s modified eagle medium (DMEM) supplemented with 10% fetal bovine serum (FBS), 100 U/mL penicillin and streptomycin at 37°C in a humidified atmosphere of 5% CO_2_.

SARS-CoV-2 early strain B.1 (hCoV-19/CHN/SYSU-IHV/2020 strain, Accession ID on GISAID: EPI_ISL_444969) was isolated from a sputum sample from a woman admitted to the Eighth People’s Hospital of Guangzhou. The SARS-CoV-2 Beta (B.1.351, SARS_CoV-2_human_CHN_20SF18530_2020, Accession ID on GWH: WHBDSE01000000) and Delta (B.1.617.2, GDPCC 2.00096) variants were isolated from patients with COVID-19 admitted in the Guangzhou Eighth People’s Hospital by Center for Disease Control and Prevention of Guangdong Province (*62*). The SARS-CoV-2 Omicron variant (B.1.1.529) was isolated from a patient with COVID-19 admitted to the Shenzhen Third People’s Hospital (*63*).

SARS-CoV-2 infection experiments were performed at the Biosafety level 3 (BSL-3) facilities of Sun Yat-sen University (Guangzhou), Guangzhou Customs District Technology Center (Guangzhou), and Shenzhen Third People’s Hospital (Shenzhen), which all possess a license for SARS-CoV-2 study approved by the China National Health Commission.

### SARS-CoV-2 replicon assays

The replicon system was reported in our previous work (*43*). The luciferase assays were performed following the manufacturer’s instructions (Promega Corporation). In brief, cells in 24-well plates were transfected with 500 ng pBAC-SARS-CoV-2-Replicon-Luciferase plasmid and 10 ng RL-TK plasmid. After 6 to 8 hours of transfection, the supernatant was discarded and replaced with fresh DMEM, followed by addition each compound (described in table S1) to the media with the final concentration of 50 μM, 10 μM, 5 μM, 2 μM, 1 μM, 0.1 μM or 0.01 μM. After 60 hours, cells were lysed in 200 μL Passive Lysis Buffer (PLB). Each lysate (20 μL) was transferred into 96-well white plate and then mixed with 20 μL Luciferase Assay Reagent II, followed by 20 μL of Stop & Glo solution. The luminescence values of the two-step reaction were recorded using a luminescence detector in Synergy H1 Hybrid Multi-Mode Reader.

### Anti-SARS-CoV-2 activity assays in cell culture

Vero E6 and Huh7 cells were seeded at 2 × 10^4^ cells per well in 48-well plates. Cells were allowed to adhere for 16 to 24 hours and then infected at multiplicity of infection (MOI) of 0.05 with SARS-CoV-2 for 1 hour at 37°C. Then, viral inoculum was removed, and cells were washed two times with pre-warmed phosphate-buffered saline (PBS). Medium containing dilutions of compounds or dimethyl sulfoxide (DMSO) was added. At 48 hours post infection, supernatants or cells were harvested for qRT-PCR analysis. The dose-response curves were plotted from viral RNA copies versus the drug concentrations using GraphPad Prism 6 software.

### qRT-PCR analysis

For SARS-CoV-2 RNA quantification, RNA was isolated by Magbead Viral DNA/RNA Kit (CWBIO). SARS-CoV-2 nucleic acid detection kit (Da’an Company) was used to detect the virus. The limit of detection (LOD) of this kit reached 0.5 copies/μL. For the detection of the viruses and cytokines in mouse tissues, total RNA was isolated from tissue samples with TRIzol per the manufacturer’s instructions. mRNA was reverse transcribed into cDNA by PrimeScript RT reagent Kit (Takara). The cDNA was amplified by a fast two-step amplification program using ChamQ Universal SYBR qPCR Master Mix (Vazyme Biotech Co., Ltd) or Taq Pro HS Universal Probe Master Mix (Vazyme Biotech Co., Ltd). *Gapdh* was used to normalize the input samples using the ΔCt method. The relative mRNA expression of each gene was normalized to *Gapdh* housekeeping gene expression in the untreated condition, and fold change was calculated by the ΔΔCT method relative to those in untreated samples. The qRT-PCR primers are listed in table S7.

### CCK-8 cell viability assay

To investigate the effect of drugs on cell viability, Vero E6 cells were seeded in 96-well plates at a density of 20,000 cells per well and were treated with drugs at indicated concentrations (0, 0.01, 0.1, 1, 5, 10, 50, 75, 100, 200 μM) for 48 hours. Cell viability was tested by using Cell Counting Kit-8 (CCK-8, Bimake, B34302). The figures were plotted from cell viability percentage versus the drug concentrations using GraphPad Prism 6 software.

### p*K*_a_ Determination

The p*K*_a_ value of test compounds were detected by using a fast-UV method with Sirius T3 Titrator. First, the blank calibration and Fast UV buffer calibration were performed where an empty sample vial was placed in the sample position. The buffer was fully titrated, and reference spectra were collected at all pHs. The sample was then detected with the following process: Methanol was used as a cosolvent to measure the psKa (cosolvent dissociation constant). Five μL of the compound stock solution of 10 mM was added to a sample vial. Three weight/volume ratios of methanol cosolvent were tested and the total starting volume for titration was set to 1.5 mL. A spectrometer detected the turbidity, and the pH range of titration by a fast UV method was 2 to 12. The titration data was obtained from a proper titration and the psKa test result was obtained by Yasuda-Shedlovsky extrapolation method for fast UV psKa method.

### Determinations of Thermodynamic Solubility

Test compounds were weighed out for measuring the solubility (about 1.5 mg each in three separate 1.5 mL glass vials). For each compound, one vial was used to do the standard and the other two were used to measure the solubility in duplicate. The proper volume of ultrapure water was added to each vial of the solubility sample plate using pipettes. One stir stick was added to each vial and the vials were sealed using a molded polytetrafluoroethylene (PTFE)/silicone plug. The solubility sample plate was transferred to an Eppendorf Thermomixer Comfort plate shaker and shaken at 25°C at 1100 rpm for 24 hours. Then the stir sticks were removed using a magnet, and the samples were transferred from the solubility sample plate into a filter plate with pipettes. All compounds were filtered under a vacuum condition. The filtrate was diluted 1000-fold with a mixture of H_2_O and acetonitrile (1:1 in v/v). The plates were vortexed for 5 min. For standards (STD), the necessary volume was calculated to dissolve each standard sample to the concentration of 1.5 mg/mL. DMSO was added to each standard sample. The standard vials were covered. The standard plate was placed on the Eppendorf Thermomixer Comfort plate shaker for 5 min at 25°C and 1100 rpm. After 5 min, each compound was completely dissolved. From the 1.5 mg/mL DMSO standard plate, 5 μL DMSO STD was transferred into the remaining empty plate, and then 5 μL ultrapure water and 490 μL of a mixture of H_2_O and acetonitrile (1:1 in v/v) was added to that plate to have a concentration of 15 μg/mL. From the 15 μg/mL standard plate, 50 μL was transferred into a new plate, and 450 μL of a mixture of H_2_O and acetonitrile (1:1 in v/v) was added to that plate to have a final standard concentration of 1.5 μg/mL. The solubility sample plate and standard plate were placed into the well plate autosampler at 4°C and evaluated by liquid chromatography-tandem mass spectrometry (LC-MS/MS) analysis (LC system: Shimadzu; MS system: Triple QuadTM 5500 instrument from AB Inc with an ESI interface). All calculations were carried out using Microsoft Excel.

### LogP Determination

The stock solutions of test compounds were prepared in DMSO to 10 mM or 30 mM. The 5 μL of stock solution (30 mM) or 15 μL of stock solution (10 mM) of each sample was placed into their proper 96-well rack. A volume of 500 μL of PBS pH 7.4 was added into each vial of the cap-less LogP plate, followed by the addition of 500 μL of 1-octanol. The assay was performed in duplicate. One stir stick was added to each vial and sealed with a molded PTFE/Silicone plug. The LogP plate was transferred to the Eppendorf Thermomixer Comfort plate shaker and shook at 25°C at 1,100 rpm for 1 hour. After incubation, the stir sticks were removed by a magnet. To separate the phases, the samples were transferred and centrifuged at 25°C at 20,000 g for 20 min. The upper (1-octanol) and lower (buffer) phases were separated and transferred to the empty tubes. Aliquots of 5 μL were taken from the upper phase, followed by 495 μL of methanol. After 1 min of a vortex, aliquots of 50 μL were taken from the diluent, followed by 450 μL of methanol. Aliquots of 50 μL were taken from the lower phase, followed by 450 μL of methanol. The samples were evaluated by LC-MS/MS analysis, and all calculations were carried out using Microsoft Excel.

### PK study in rat

Male Sprague Dawley rats (180 to 220 g, n=3) were fasted for 12 hours before drug administration. ATV006 was administered IV at 5 mg/kg or orally at 25 mg/kg. Blood samples were collected from the jugular vein into anticoagulant EDTA-K2 tubes at 0.083, 0.25, 0.5, 1, 2, 3, 4, 6, 8 and 24 hours for the IV group, and 0.25, 1, 0.5, 2, 3, 4, 6, 8 and 24 hours for the oral group, respectively. All samples were centrifuged under 4000 rpm for 10 min at 4°C and the plasma samples were collected and stored at -65°C for future analysis. An aliquot of 50 μL each plasma sample was treated with 250 μL of acetonitrile. The samples were centrifuged under 4000 rpm for 10 min and filtered through 0.2 μm membrane filters. The concentrations of analytes in each sample were analyzed by LC-MS/MS.

### PK study in cynomolgus monkeys

Three male cynomolgus monkeys (2 to 5 years of age, weighing 3 to 5 kg) were orally administrated AVT006 of 10 mg/kg on day 1. Blood samples for plasma were collected from a jugular vein into anticoagulant EDTA-K_2_ tubes at 0.083, 0.25, 0.5, 1, 2, 4, 8, 24 and 48 hours after administration. After a 3-day washout period, each animal was administered IV with ATV006 at a dose of 5 mg/kg, followed by blood collection from the jugular vein at the specified time points. All samples were centrifuged under 4000 rpm for 10 min at 4°C and the plasma was collected and stored at -65°C for future analysis. The concentration of analytes in each sample were analyzed by LC-MS/MS.

### Tissue distribution study in rats

Eighteen Sprague Dawley rats were randomly divided into two groups (n = 9) and each group received a single oral administration of either 100 mg/kg ATV006 or 100 mg/kg GS-441524. Three rats in each group were anesthetized at 1-, 4-, and 24-hours post-dosing. Blood samples were collected into the heparinized tubes which containing PhosSTOP and DTNB and centrifuged. Tissue samples, including liver, kidney, and lung were harvested after perfusion with saline and immediately homogenized for further analysis as previously described (*64*). Tissue samples were individually homogenized with four equivalent volumes of H_2_O containing PhosSTOP EASYpack (PhosSTOP) (Roche) and 5,5′-dithiobis (2-nitrobenzoic acid) (DTNB, ≥ 98%) (Sigma-Aldrich). For GS441524 determination, 40 μL plasma or homogenate was immediately added to 200 μL acetonitrile-methanol (50/50, v/v) which containing 50 nM Nadolol, vortexed thoroughly and centrifuged at 4°C. For RMP and RTP determination, 50 μL of plasma or homogenate was immediately added to 150 μL of 2% formic acid and 5 μL of 10 μM ^13^C,^15^N-adenosine triphosphate (ATP) aqueous solution, then vortexed thoroughly and centrifuged at 4°C. Supernatant in a volume of 180 μL was mixed with an equivalent volume of water for solid-phase extraction (SPE) with a weak anion exchange plate (Waters Oasis WAX μElution plate). Finally, the eluate was gently evaporated to dryness under a gentle stream of air, and the residue was reconstituted with 100 μL H_2_O for analysis on a Waters Positive Pressure-96 processor.

### Tissue distribution study in mice

Five C57BL/6 mice were fasted for 12 to 16 hours before oral administration with a single dose of 100 mg/kg ATV006. After 1 hour of administration, 0.5 mL of the blood sample was taken from heart. Liver, kidney, lung, and brain tissues were harvested. The blood samples were processed to the same method as in the rat PK studies. Tissue samples were homogenized and extracted with 70% methanol. After centrifugation at 4000 rpm for 10 min, the supernatants were transferred to a clean tube and the concentrations of analytes in each sample was analyzed by LC-MS/MS.

### Mouse models of SARS-CoV-2 infection

Three different mouse models were used in this study, including mice with Ad5-delivered hACE2 (Ad5-hACE2), knock-in hACE2 at the *mAce2* gene locus (KI-hACE2) and transgenic K18-hACE2 (*49*, *50*, *53*). For establishment of Ad5-hACE2 model, 6 week old BALB/c mice, which were purchased from Human SJA Laboratory Animal Co., Ltd (Changsha, China), were lightly anesthetized with isoflurane and transduced intranasally with 2.5 × 10^8^ focus-forming units (FFU) of Ad5-ACE2 in 75 μL DMEM. Five days post transduction, mice were infected intranasally with SARS-CoV-2 [1 × 10^5^ plaque-forming units (PFU)] in a total volume of 50 μL DMEM as previously described (*50*). The hACE2 knock-in (KI-hACE2) mice (strain NF11031 on C57BL/6 background) were purchased from the Division of Animal Model Research, Institute for Laboratory Animal Resources, National Institutes for Food and Drug Control, Beijing, China. The mice (age of 5.5 weeks) were infected intranasally with SARS-CoV-2 (2 × 10^5^ PFU). K18-hACE2 transgenic mice (Jax strain 034860) were purchased from Jackson Lab through iBio Logistics Co., Ltd. The mice (age of 8 to 10) weeks were infected intranasally with SARS-CoV-2 (1 × 10^3^ or 5 × 10^2^ PFU).

The mouse SARS-CoV-2 infection experiments were performed at the BSL-3 facilities of Sun Yat-sen University and Guangzhou Customs District Technology Center, which obtained a license for small animal study of SARS-CoV-2 approved by the China National Health Commission. All animal study protocols were approved by the Institutional Animal Welfare Committee and all procedures used in animal studies complied with the guidelines and policies of the Animal Care and Use Committee of the respective research units.

### SARS-CoV-2 plaque assay

Vero E6 cells were grown in 12-well plates to 70 to 80% confluence and infected with 500 μL of medium containing viruses at indicated dilutions. After 1 hour at 37°C, the inoculate were removed. 1 mL of 0.8% agar (Amresco) in DMEM with 2% FBS was overlaid onto cells. Plaques were analyzed at 72 hours. For plaque staining, 1 mL of 70% ethanol containing 0.5% purple crystal (Sigma-Aldrich) was overlaid onto cells. 24 hours later, the stained plaques were counted.

### Focus forming assay (FFA)

Vero E6 cells were seeded in 96-well plates one day before infection. Tissue homogenates were serially diluted and used to inoculate Vero E6 cells at 37°C for 1 hour. Inoculate were then removed before adding 125 μL of 1.6% carboxymethylcellulose warmed to 37°C per well. After 24 hours, cells were fixed with 4% paraformaldehyde and permeabilized with 0.2% Triton X-100. Cells were then incubated with a rabbit anti-SARS-CoV-2 nucleocapsid protein polyclonal antibody (Cat. No.: 40143-T62, Sino Biological), followed by a horseradish peroxidase (HRP)-labeled goat anti-rabbit secondary antibody (Cat. No.: 109-035-088, Jackson Immuno Research Laboratories). The foci were visualized by TrueBlue Peroxidase Substrate (KPL) and counted with an ELISPOT reader (Cellular Technology). Viral titers were calculated per gram of tissue.

### Hematoxylin and eosin staining and immunohistochemistry.

For hematoxylin and eosin (H&E) staining, mouse lung and spleen sections were fixed in zinc formalin and embedded with paraffin. Tissue was sectioned to slices of about 4 μm and were stained with H&E. For immunohistochemistry, slides were blocked in 1% bovine serum albumin for 1 hour and stained with a primary antibody to SARS-CoV-2 S protein (GeneTex, Cat No. GTX632604) in blocking buffer at 4°C overnight. The slides were incubated with the biotinylated-secondary antibodies (Jackson ImmunoResearch Laboratories Inc.715-065-151) for 30 min at room temperature. The slides were then incubated with HRP-conjugated streptavidin (Jackson ImmunoResearch Laboratories Inc.016-030-084) for 30 min at room temperature in a dark humid box and subsequently stained with 3,3′-Diaminobenzidine (DAB, Vector Laboratories, SK-4100). Counterstaining with hematoxylin (Beijing Leagene Biotech) and mounting with neutral resin (Solarbio) was performed. Finally, the images were scanned by NanoZoomer S360 (Hamamatsu Photonics).

### Statistical analysis.

All values are presented as mean ± SD of individual samples. Data analysis was performed with GraphPad Prism Software (GraphPad Software Inc., version 6.01). A Shapiro-Wilk test was utilized for analyzing normality distribution. A Log-Rank test, one-way analysis of variance (ANOVA) with Dunnett’s correction for multiple comparisons, or Kruskal-Wallis test with Dunn's correction for multiple comparisons was utilized for statistical analysis and respective details are indicated in figure legends (Fig. 4 to 6). P-values < 0.05 were considered statistically significant.

## Supplementary Materials

### Other Supplementary Material for this manuscript includes the following:

MDAR Reproducibility Checklist
