# Supplementary material for: The adenosine analog prodrug ATV006 is orally bioavailable and has preclinical efficacy against parental SARS-CoV-2 and variants
Source: Sci Transl Med. 2022 May 17:eabm7621. doi: 10.1126/scitranslmed.abm7621 (PMC9161374; doi:10.1126/scitranslmed.abm7621)
Supplement: Supplementary file 1 — Figs. S1 to S6 Tables S1 to S7 [file scitranslmed.abm7621_sm.pdf]

Science Translational Medicine
AAAS

Supplementary Materials for

The adenosine analog prodrug ATV006 is orally bioavailable and has preclinical efficacy against parental SARS-CoV-2 and variants

Liu Cao *et al.*

Corresponding authors: Deyin Guo, guodeyin@mail.sysu.edu.cn; Xumu Zhang, zhangxm@sustech.edu.cn; Jincun Zhao zhaojincun@gird.cn; Yingjun Li, liyj@sustech.edu.cn
DOI: 10.1126/scitranslmed.abm7621

The PDF file includes:

Figs. S1 to S6
Tables S1 to S7

Other Supplementary Material for this manuscript includes the following:

MDAR Reproducibility Checklist
Data file S1

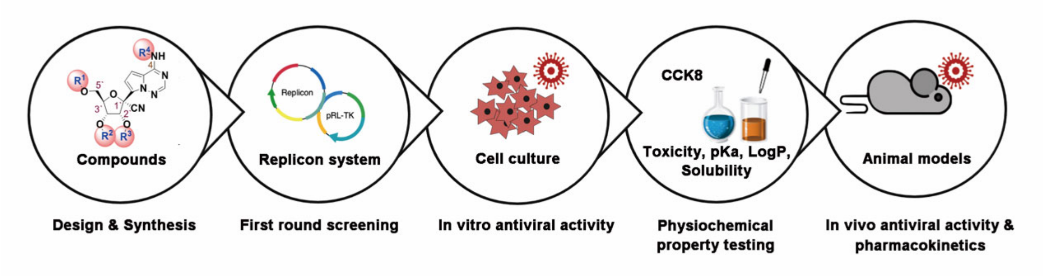

Fig. S1. A flow chart for selecting candidate compounds for further testing.

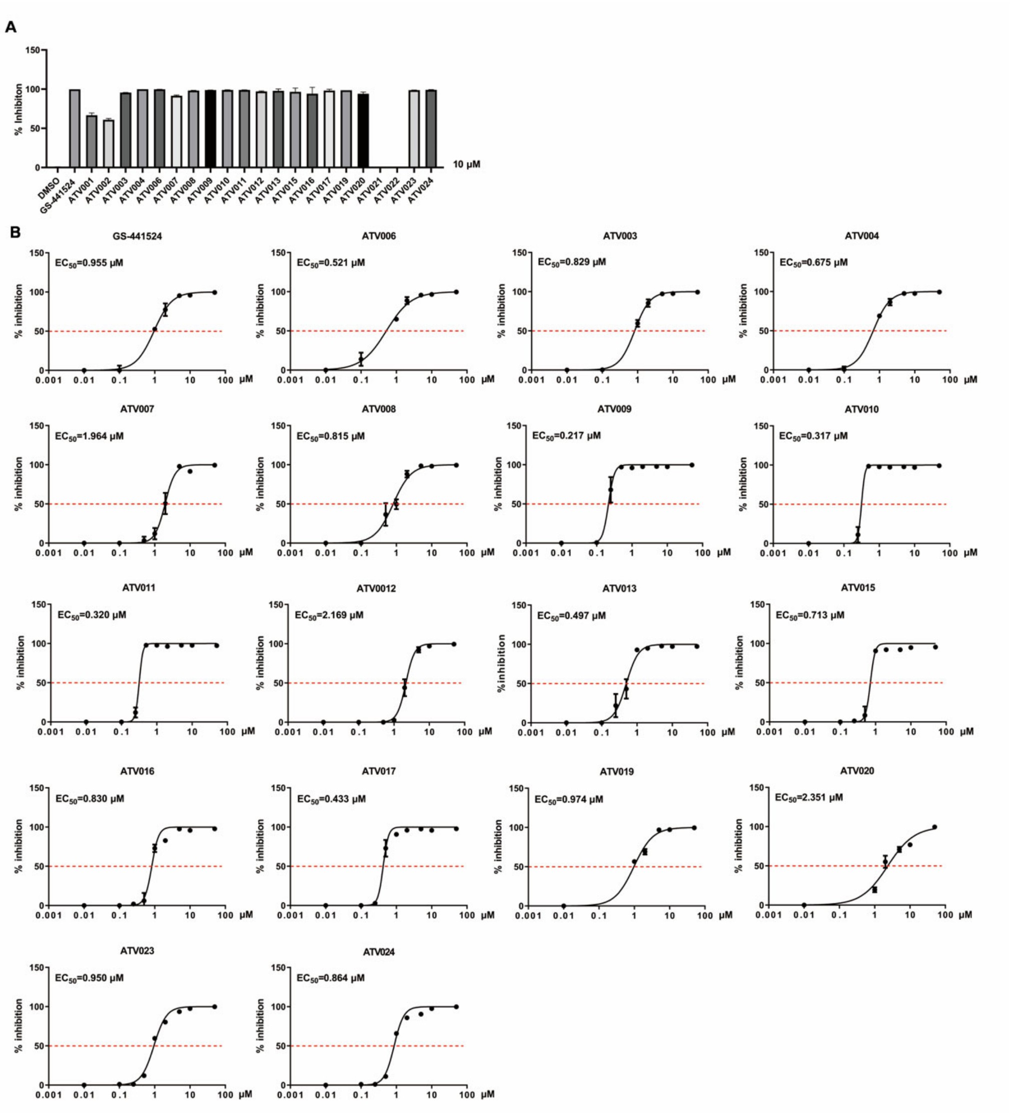

Fig. S2. Antiviral activity of 21 compounds in SARS-CoV-2 replicon system.

(**A**) HEK 293T cells transfected with SARS-CoV-2-Rep-Luci were treated with dimethyl sulfoxide (DMSO) or one of 21 compounds at 10 μM. At 60 hours post-transfection, the cells were analyzed by a luciferase reporter assay. (**B**) Each indicated compound was evaluated to determine the concentration for 50% of maximal effect (EC_{50}), which is indicated above each plot and shown by the red dashed line. Data are presented as mean ± SD.

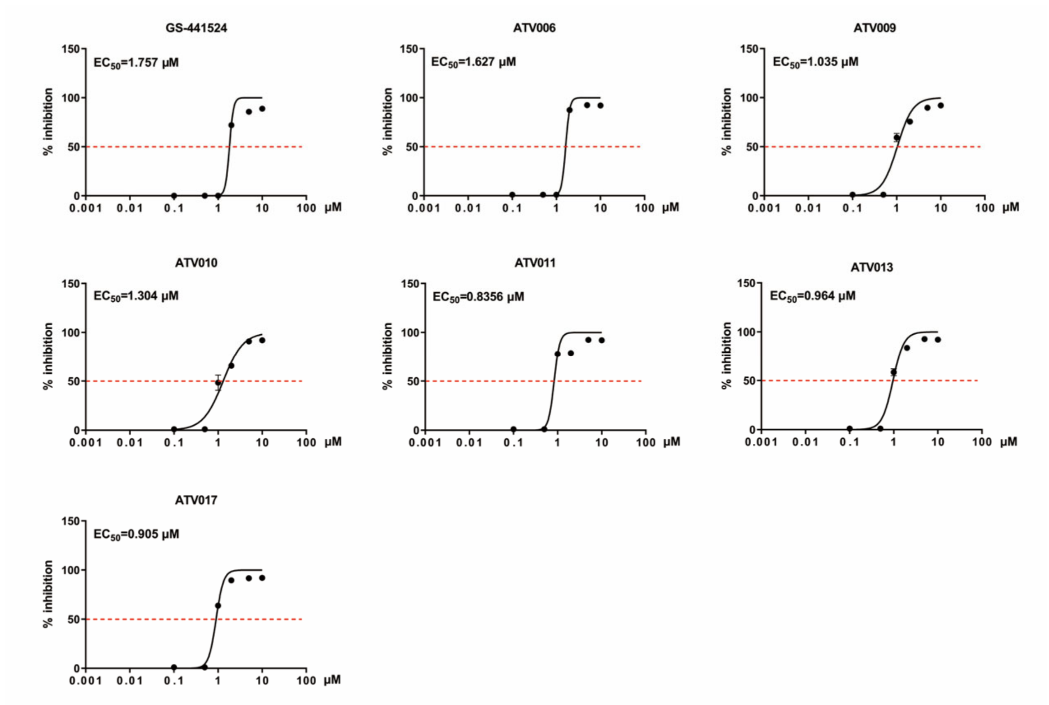

Fig. S3. Antiviral activity of compounds against SARS-CoV-2 (B.1) in Huh7 cells. Huh7 cells were infected with the B.1 strain of SARS-CoV-2 at a multiplicity of infection (MOI) of 0.05 and treated with dilutions of each indicated compound (0, 0.01, 0.1, 0.5, 1, 2, 5 and 10 μM) for 48 hours. Viral yield in the cultured supernatant was then quantified by Quantitative real-time polymerase chain reaction (qRT-PCR) [% inhibition = (1-compound virus copies/ control group virus copies) × 100]. The EC_{50} values for each compound were determined, which are indicated above each plot and shown by the red dashed line. Data are presented as mean ± SD.

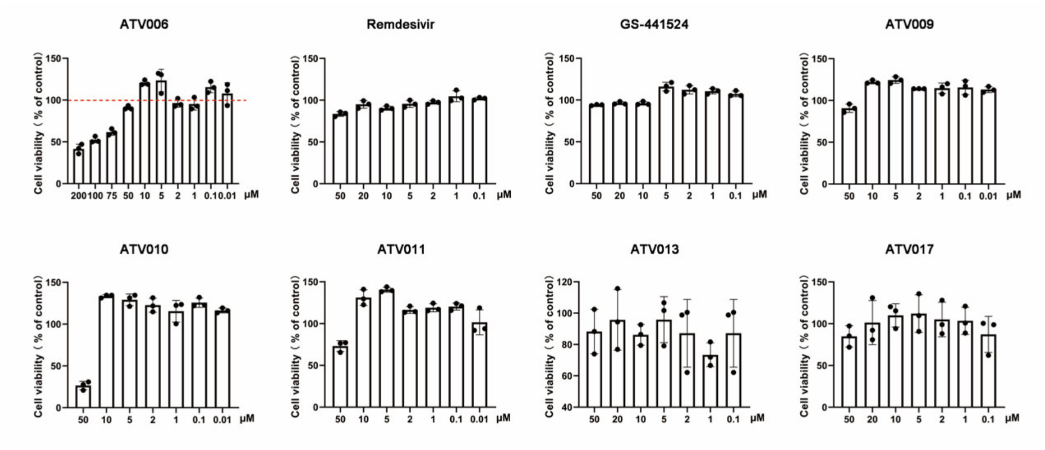

Fig. S4. Cytotoxicity assay of compounds in Vero E6 cells.

Vero E6 cells were plated in 96-well plates and treated with increasing concentrations of each indicated compound, ranging from 0 to 200 µM, for 48 hours. Cell viability was tested using Cell Counting Kit-8 (CCK-8). Data are presented as mean ± SD.

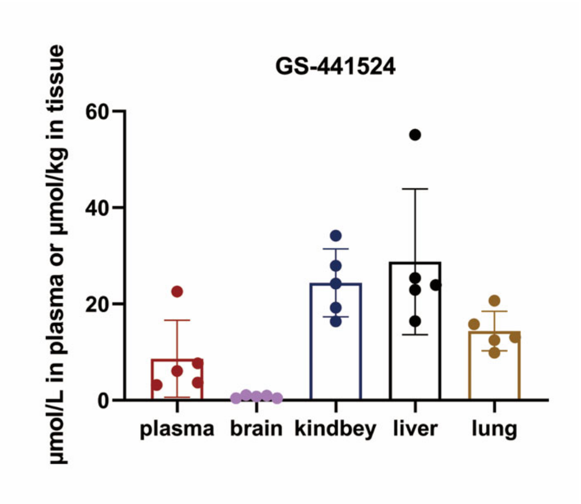

Fig. S5. The tissue distribution of ATV006 in C57BL/6 mice.

Tissue distribution of GS-441524 is shown for plasma, brain, lung, liver, and kidney after oral administration of 100 mg/kg ATV006 to C57BL/6 mice (n = 5, mean ± SD).

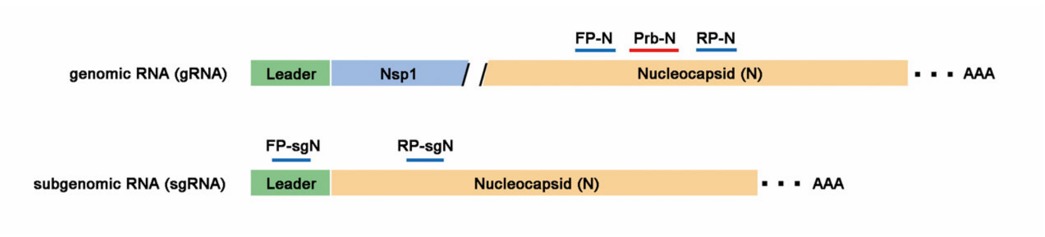

Fig. S6. Diagram of the genomic RNA (gRNA) and subgenomic RNA (sgRNA) structures of SARS-CoV-2.

The target positions of the primer/probe set of genomic RNA (gRNA) and subgenomic RNA (sgRNA) of SARS-CoV-2 are shown. In this study, only forward primers (FP) and reverse primers (RP) were used to detect sgRNA of SARS-CoV-2, and no probe (Prb) was used. The sequences of primer and probe sets are listed in table S7. Nsp1, nonstructural protein 1.

Table S1. Anti-SARS-CoV-2 replicon activity and data analysis of adenosine analogue prodrugs.

Compound	EC_{50} *(Confidence 95%)	EC_{90} *(Confidence 95%)
GS-441524	0.955 (0.808-1.127)	3.252 (2.129-5.666)
ATV006	0.521 (0.406-0.668)	3.225 (1.849-5.625)
ATV003	0.829 (0.704-0.976)	2.315 (1.480-3.620)
ATV004	0.675 (0.558-0.817)	2.067 (1.488-2.873)
ATV007	1.964 (1.723-2.238)	3.728 (2.422-5.738)
ATV008	0.815 (0.665-0.997)	3.137 (1.497-6.575)
ATV009	0.217 (0.201-0.234)	0.313 (0.275-0.357)
ATV010	0.317 (0.285-0.350)	0.306 (very wide)
ATV011	0.320 (0.296-0.346)	0.384 (0.309-0.476)
ATV012	2.169 (1.987-2.368)	3.511 (2.409-5.117)
ATV013	0.497 (0.429-0.575)	1.139 (0.699-1.854)
ATV015	0.713 (0.678-0.750)	0.962 (0.914-1.013)
ATV016	0.830 (0.799-0.862)	1.229 (1.184-1.277)
ATV017	0.433 (0.380-0.493)	0.576 (0.478-0.696)
ATV019	0.974 (0.6562-1.446)	3.814 (1.116-13.030)
ATV020	2.351 (1.902-2.906)	13.390 (5.090-35.230)
ATV023	0.950 (0.830-1.088)	2.014 (1.385-2.929)
ATV024	0.864 (0.7513-0.9937)	1.472 (1.122-1.932)

* denotes μM

Table S2. Permeability and efflux ratio determination of GS-441524, ATV006, ATV019, and ATV020 in Caco-2 cells.

Compound	Caco-2 AB/BA (Papp (10^{-6} cm/s)) [a]	Efflux ratio
GS-441524	1.22/1.20	0.98
ATV006	0.51/0.87	1.7
ATV019	0.28/0.68	2.47
ATV020	0.17/0.22	1.28

[a] Papp (A to B) < 2, low permeability; 2 < Papp (A to B) < 10, moderate permeability; Papp (A to B) > 10, high permeability.

Table S3. Anti-SARS-CoV-2 variant activity and data analysis of adenosine analogue prodrugs in Vero-E6 cells.

Compound	EC_{50} *(Confidence 95%)	EC_{90} *(Confidence 95%)
Remdesivir (B.1)	2.279 (2.232-2.326)	4.250 (3.950-4.572)
Remdesivir (Beta, B.1.351)	1.780 (1.573-2.015)	5.016 (3.312-7.598)
Remdesivir (Delta, B.1.617.2)	1.645 (1.285-2.1.6)	7.342 (2.868-18.800)
Remdesivir (Omicron, B.1.1.529)	1.238 (1.024-1.497)	5.691 (1.936-16.730)
GS-441524 (B.1)	1.709 (1.665-1.775)	2.392 (2.305-2.481)
GS-441524 (Beta, B.1.351)	1.354 (1.329-1.379)	2.316 (2.219-2.417)
GS-441524 (Delta, B.1.617.2)	0.957 (0.909-1.008)	1.355 (1.042-1.763)
GS-441524 (Omicron, B.1.1.529)	0.190 (0.181-0.199)	0.363 (0.353-0.373)
ATV006 (B.1)	1.360 (1.299-1.422)	2.180 (1.996-2.381)
ATV006 (Beta, B.1.351)	1.127 (0.946-1.343)	1.358 (0.382-4.828)
ATV006 (Delta, B.1.617.2)	0.349 (0.329-0.368)	0.506 (0.468-0.547)
ATV006 (Omicron, B.1.1.529)	0.106 (0.103-0.110)	0.269 (0.265-0.272)
ATV009 (B.1)	1.329 (1.277-1.383)	1.526 (very wide)
ATV009 (Beta, B.1.351)	1.484 (1.468-1.500)	1.781 (1.773-1.789)
ATV009 (Delta, B.1.617.2)	0.492 (0.464-0.523)	0.678 (0.492-0.934)
ATV010 (B.1)	0.696 (0.635-0.763)	1.199 (0.976-1.473)
ATV010 (Beta, B.1.351)	1.002 (0.993-1.011)	1.295 (1.006-1.666)
ATV010 (Delta, B.1.617.2)	0.457 (0.419-0.493)	0.619 (0.510-0.752)
ATV011 (B.1)	2.117 (0.846-5.298)	2.636 (very wide)
ATV011 (Beta, B.1.351)	2.302 (2.059-2.573)	2.947 (1.335-6.503)
ATV011 (Delta, B.1.617.2)	0.408 (0.368-0.453)	0.842 (0.676-1.045)
ATV013 (B.1)	2.262 (0.964-5.311)	2.866 (very wide)

ATV013 (Beta, B.1.351)	2.434 (1.969-3.010)	3.090 (0.957-9.984)
ATV013 (Delta, B.1.617.2)	0.965 (0.925-1.007)	1.414 (1.202-1.662)
ATV017 (B.1)	2.188 (0.723-6.622)	2.482 (very wide)
ATV017 (Beta, B.1.351)	2.847 (2.415-3.356)	3.405 (1.817-6.382)
ATV017 (Delta, B.1.617.2)	0.428 (0.303-0.606)	0.516 (0.449-0.593)

* denotes μM

Table S4. Anti-SARS-CoV-2 activity and data analysis of adenosine analogue prodrugs in Huh7 cells.

Compound	EC_{50} *(Confidence 95%)	EC_{90} *(Confidence 95%)
GS-441524	1.757 (1.408-2.193)	2.078 (very wide)
ATV006	1.627 (1.268-2.089)	1.955 (very wide)
ATV009	1.035 (0.841-1.275)	1.311 (0.993-1.731)
ATV010	1.304 (1.054-1.613)	2.741 (1.445-5.200)
ATV011	0.836 (0.652-1.071)	1.012 (0.895-1.145)
ATV013	0.964 (0.847-1.098)	1.324 (1.083-1.619)
ATV017	0.905 (0.818-1.002)	1.173 (1.005-1.368)

* denotes μM

Table S5. The pKa, LogP, and solubility testing results of ATV006

Compound ID	pKa Value (Basic)	LogP Value	Solubility (μg/mL)
ATV006	3.66	0.86	686.02

Table S6. Changes in cytokine transcripts after infection.

	(SARS-CoV-2 K18-hACE2 mouse model-lung) Vehicle/ATV006 fold change + standard error of the mean	P
Ifnb	5.85±4.59	0.1963
Ifng	25.56±17.77	0.0605
Cxcl10	43.19±20.74	0.1008
Ccl2	24.69±25.14	0.2245
Il6	3.14±2.37	0.7137
Il1β	0.66±0.01	0.4484

Table S7. qPCR primers (P) and probes (Prb) used for detection of viral genomes and indicated genes.

Gene		Sequence (5‘-3’)
DA’AN SARS-COV-2-N	FP	AAGAAATTCAACTCCAGGCAGC
	RP	GCTGGTTCAATCTGTCAAGCAG
	Prb	TCACCGCCATTGCCAGCCA
SARS-COV-2 sgN	FP	CCAGGTAACAAACCAACAA
	RP	TGAGTGAGAGCGGTGAACCAA
Gapdh	FP	AGAACATCATCCCTGCATCC
	RP	CACATTGGGGGTAGGAACAC
Il6	FP	AACCAAGAGATAAGCTGGAGTCAC
	RP	AACGCACTAGGTTTGCCGAG
Il1b	FP	TGCCACCTTTTGACAGTGATGA
	RP	ATCAGGACAGCCCAGGTCAA
Cxcl10	FP	TGCAGGATGATGGTCAAGCC
	RP	CCACTTGAGCGAGGACTCAG
Ifng	FP	CAGCAAGGCGAAAAAGGATGC
	RP	CTTCCTGAGGCTGGATTCCG
Ifnb	FP	GTGGGAGATGTCCTCAACTGC
	RP	TCTCTGCTCGGACCACCATC
Ccl2	FP	TGGGCCTGTTGTTCACAGT
	RP	TTCTCCAGCCGACTCATTG
